# Environmental enrichment influences the relationship between lateralization and neophobia in zebrafish larvae

**DOI:** 10.1007/s10071-025-02025-1

**Published:** 2025-12-24

**Authors:** Gabriela Gjinaj, Elia Gatto, Marco Dadda

**Affiliations:** 1 Department of General Psychology, University of Padova, Via Venezia 8, 35131 Padova, Italy; 2 Department of Chemical Pharmaceutical and Agricultural Sciences, University of Ferrara, Via Luigi Borsari 46, 44121 Ferrara, Italy; 3 Department of Life Sciences and Biotechnology, University of Ferrara, Via Luigi Borsari 46, 44121 Ferrara, Italy

**Keywords:** Environmental enrichment, Lateralization, Neophobia, Phenotypic plasticity, Zebrafish larvae

## Abstract

Neophobia, the aversion to novelty, is a conserved survival trait that influences individuals’ decision-making. In many species, juveniles showed less avoidance of novelty than adults, likely due to their limited experience and their need to gather environmental information. A challenging question is how environmental factors shape sensory processing and behavioural responses. Due to the rapid development of their nervous system and high sensitivity to environmental conditions, zebrafish (*Danio rerio*) represent a valuable model for investigating the phenotypic plasticity of neophobia. Previous studies showed that two-week-old larvae are more inclined to explore novel stimuli, while older larvae exhibit neophobic responses, indicating an age-dependent shift in exploratory behaviour. Lateralization, i.e. the specialization of the left and right side of the brain to attend different sensory information, may modulate the neophobic response. Here, we investigated how environmental experience alters the relationship between neophobia response and asymmetrical visual inspection in zebrafish larvae throughout early development. After hatching, larvae were reared in either enriched or barren environments. Neophobic response was assessed at 7, 14, and 21 days post-fertilization (dpf) using a novel object test. Results showed that 21dpf larvae raised in enriched environments approached the novel stimulus more quickly and frequently than barren larvae. Enrichment also significantly influenced lateralization, with enriched larvae displaying stronger lateralization. Notably, in enriched larvae, individuals that inspected the novel object more frequently showed weaker and less consistent lateralization. Results suggest that early environmental enrichment affects not only neophobic behaviour, but also the development of lateralized cognitive processing.

**Supplementary Information:**

The online version contains supplementary material available at 10.1007/s10071-025-02025-1.

## Introduction

Neophobia, the aversion or avoidance of novelty, is a conserved behavioural trait observed across a wide range of taxa and plays a critical role in individuals survival by minimizing exposure to potential threats (Greggor et al. 2015). Neophobia is underpinned by neuroendocrine and neural processes involving stress-related pathways and neurotransmitter systems (Koolhaas et al. 2011; Carter et al. 2013). However, the contribution of no-genetic factors, i.e. environmental condition, during the life of an individual, such as early-life stress and social environment, can calibrate neural circuits regulating neophobic response (Boogert et al. 2006; Stamps and Groothuis 2010).

As a consequence, neophobia can directly shape foraging strategies, predator–prey interactions, and habitat use, influencing individual fitness and population dynamics. For example, a strong neophobic response may reduce predation risk but at the cost of missed opportunities to exploit novel resources (Greenberg 2003; Brown et al. 2007). Conversely, reduced neophobia can facilitate behavioural flexibility and innovation (Greggor et al. 2015; Guido et al. 2017). On the other hand, we might expect that individuals with less experience would be more likely to be attracted to novelty (Eliassen et al. 2007; Crane and Ferrari 2017). Indeed, younger individuals tend to forage longer and search for new resources than adults, mainly due to the higher cost of offspring and territory losses for the older stages (Sullivan 1988; Ralls and Siniff 1990). For example, Bali myna birds show age-dependent differences in neophobia, with adults demonstrating greater avoidance of novel objects than juveniles (Miller et al. 2022). Similarly, in reptiles hesitation in approaching unfamiliar prey has been reported, indicating a broad taxonomic prevalence of neophobic behaviour (Szabo and Ringler 2023). This scenario suggests a positive correlation between age and the development of neophobic response (adaptivity hypothesis; Mata et al. 2013).This theoretical framework has found both support (Bergman and Kitchen 2009; Katwaroo-Andersen et al. 2016; O’Hara et al. 2017) and contradiction in the literature (Joyce et al. 2016; Greggor et al. 2020 ), indicating that this phenomenon remains poorly understood. It is likely that multiple factors converge in shaping how individuals respond to novelty and acquire information from experiences.

In recent years, increasing attention has been devoted to the role of hemispheric specialization in shaping behavioural responses to novelty and potential threats across a range of animal species. In teleost fish cerebral lateralization has been shown to influence individual behavioural strategies such as exploration, boldness, and fear responses (Dadda et al. 2007; Reddon and Hurd 2009). These lateralized patterns of neural processing are evolutionarily conserved and allow animals to allocate specific cognitive tasks to distinct brain hemispheres, enhancing efficiency in environmental interaction (Vallortigara and Rogers 2005). Within this framework, several studies suggest that lateralization may modulate how individuals differently cope with their environment (Reddon and Hurd 2009; Goursot et al. 2019; Tomassetti et al. 2019), thus shaping the expression of neophobia. Specifically, right-hemisphere dominance, often expressed as a preference for using the left eye when viewing novel or potentially threatening stimuli, has been linked to increased caution and risk aversion in the domestic chick (Vallortigara and Andrew 1994).Fish displaying left-hemisphere dominance (right-eye preference), usually involved in controlling action-oriented behaviours towards visual stimuli (Vallortigara 2000; Rogers et al. 2013), may be more prone to explore unfamiliar environments, a trait associated with bolder behavioural phenotypes (Reddon and Hurd 2009; Dadda et al. 2009). Conversely, zebrafish showed a strong left-eye preference when inspecting familiar and unfamiliar objects, suggesting hemispheric specialization for familiarity (Sovrano et al. 1999; Sovrano and Andrew 2006; Rogers et al. 2013). Moreover, strongly lateralized individuals appear to be more efficient in their capacity to allocate attentional resources across simultaneous tasks supporting the hypothesis that functional cerebral asymmetries allow individuals to cope with divided attention (Dadda and Bisazza 2006).

In zebrafish (*Danio rerio*), neophobic responses are increasingly studied to understand how environmental conditions modulate cognition and behaviour. Recent work has shown that environmental context significantly influences neophobia in zebrafish. Gatto and colleagues (2022) compared larvae raised in either an enriched or barren environment at 14 days post-fertilization (hereafter dpf). Considering the tendency in zebrafish larvae to explore a novel object over a familiar one, the authors found that larvae from the barren environment spent more time exploring the familiar stimuli, whereas larvae bred in an enriched environment explored both familiar and novel stimuli almost equally. Thapa et al. (2024) demonstrated that zebrafish exposed to high-risk conditions, simulated through chemical alarm cues from injured conspecifics, exhibited enhanced anxiety-like behaviours, such as increased bottom-dwelling and reduced exploratory behaviour, in novel environments. These fish also displayed stronger neophobic responses to unfamiliar predator odors, supporting the view that neophobia can be adaptively upregulated in threatening contexts. In zebrafish, the ability to flexibly modulate neophobic responses based on environmental cues not only highlights their cognitive plasticity but also positions them as a promising model for exploring the neurobiological and ecological underpinnings of risk-sensitive behaviour.

The aim of the present study was twofold. The first was to further investigate the modulation of the neophobic response during early developmental stages in zebrafish. Zebrafish larvae represent a highly valuable model organism in neuroscience, developmental biology, and behavioural research due to their unique combination of genetic tractability and rapid development (Kalueff et al. 2014; Stewart et al. 2014). From early developmental stages, zebrafish larvae exhibit a rich behavioural repertoire (Kalueff et al. 2014). Their small size and transparency allow for high-resolution imaging of neural circuits and real-time monitoring of brain activity (Ahrens et al. 2013). Furthermore, the zebrafish genome shares substantial homology with that of humans, and many key genes involved in neurodevelopment and psychiatric disorders are conserved, making it a relevant model for translational research (Howe et al. 2013). Their early sensitivity to environmental stimuli also makes them ideal for investigating the effects of experience, enrichment, and stress on behaviour and cognition during development (Stewart et al. 2015).

Starting from the database of Gatto and colleagues (2022), we reanalysed the video recordings to obtain new behavioural measures. In their original study, Gatto and colleagues (2022) implemented a standard novel object exploration test to measure the effects of environmental enrichment on zebrafish anxiety-like behaviour by evaluating differences in behavioural response in larvae raised in either enriched environment or control barren condition. The anxiety-like behaviour has been described considering the time spent by larvae towards the novel stimulus, the number of approaches to the sector that housed the novel stimulus, and the swimming velocity. Secondly, the authors investigate the effect of early-exposition to enriched environment during ontogeny by comparing larvae at the age of 7, 14, and 21dpf.

Here we conducted a detailed scoring of the video recordings by considering new behavioural measures: inspections (approaches) toward the stimulus, the first approach to the stimulus, and the number of complete rotational inspections of the stimulus.

Secondly, we aimed at investigating whether the neophobic response was influenced by the degree of hemispheric specialization. Several studies suggest a possible link between lateralization, personality traits, and the tendency to explore unfamiliar objects or environments. In particular, it has been shown that individuals classified as bold exhibit a high degree of lateralization and are more likely to explore unfamiliar environments (Reddon and Hurd 2009; Berlinghieri et al. 2024, 2025).

Based on this evidence, the novelty of the present study concerned the effect of environment on the relationship between neophobic response and lateralization. Secondly, we evaluated how this relationship could change during development. The same set of videorecording by Gatto et al. (2022) were then used to measure lateralization in eye use during close inspection of the novel stimulus, a behavioural response not considered in the previous study. Our hypothesis was that subjects might show a preference for using either the left or right eye during close inspection of the novel stimulus. Furthermore, we hypothesized that environmental conditions may influence how individuals perceive novel objects at early developmental stages. Specifically, we aimed to assess whether larvae raised in enriched or barren environments differ in the way lateralization interacts with neophobic behaviour.

## Materials and methods

### Ethics

The treatment conditions and the following behavioural observations were collected and used in an earlier study (Gatto et al. 2022). The present study aimed to explore whether behavioural lateralization and neophobic responses are related, a question that was not investigated in the previous study by Gatto and colleagues. To do so, a completely blind experimenter (G.G.) re-analysed all the collected video recordings using a different methodological approach specific to the aim of the present studies; thus, the presented results represent novel findings. Experiments were in accordance with the law of our country (Italy, D.L. 4 Marzo 2014, n. 26) and were approved by the Ethical Committees of the University of Padova (protocol no. 61/2018) and University of Ferrara (protocol no. TLX-2019-1).

### Subjects

In the present study, a total of 120 wild-type zebrafish larvae were used. Larvae were obtained from spontaneous group spawning of adult zebrafish maintained at the Animal Behaviour and Cognition Laboratory, University of Padova. Adult zebrafish were maintained in mixed sex groups into a 150-L glass tank provided with natural gravel, vegetation and mechanical filter. A central conditioning allowed maintaining a constant water temperature 28 ± 1 °C. Fluorescence lamps placed 50 cm above the tank provided illumination with a 14:10 light: dark photoperiod. Feeding schedule was three times per day and ad libitum consisting of live *Artemia salina* nauplii (Ocean Nutrition, USA) and commercial flakes (sera Gmbh, Heinsberg, DE).

Breeders were selected the night before the start of the experiment from the maintained tank and placed into the standard breeding aquaria (Tecniplast, Italy). Two males and two females were kept overnight in the tank but separated by a transparent partition. We prepared 5 breeding tanks by placing adult zebrafish from different maintenance aquaria to reduce potential inbreeding effect that might affect behavioural response. One hour after the light on, the partition was removed, and fish were allowed to interact. After spawning (0-day post fertilization, dpf), fertilized eggs were collected and place in group (approx. 30 eggs) into Petri dishes (∅: 10 cm; h: 1.5 cm) filled with E3 medium (see Westerfield 2007 for the chemical composition) with 0.1 mL methylene blue to prevent mould growth and for its antifungal action. We performed two replicates of the spawning events with a month interval.

**Fig. 1:**
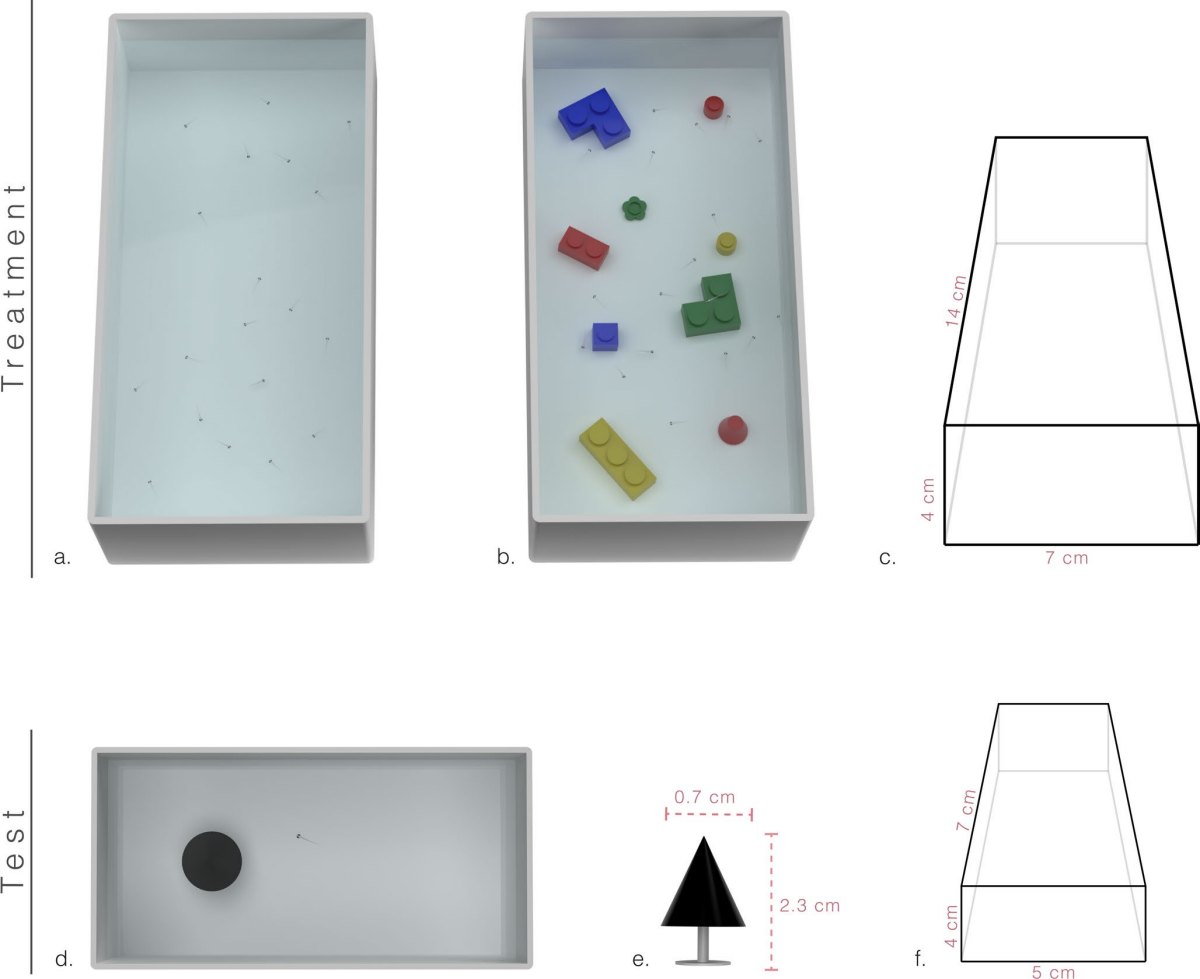
3D representations of treatment tanks (a, b) and the experimental apparatus (d). **a** Barren condition. **b** Enriched condition. **c** Schematic representation of treatment tanks with apparatus dimensions. **d** Test apparatus. **e** Stimulus used in the test apparatus. **f** Schematic representation of the test apparatus with tank dimensions

From the initial pool of larvae hatched at 4dpf, subjects were transferred to rearing tanks: 14 × 4 × 7 cm white Polylactic Acid (PLA) material box filled with 240 mL of 1x Fish Water (see Westerfield 2007). Larvae were randomly assigned to one of two treatment conditions: enriched or barren environment. In the enriched condition, rearing tanks were provided with nine Lego ^®^ bricks varying in colour (e.g., red, blue, green, yellow; Fig. 1b) and shape (e.g., cylinder, cube, L-shape, parallelepiped, flower), providing visual and tactile enrichment. In the barren condition, larvae were reared in identical tanks but without the enrichment (Fig. 1a). All larvae were maintained at the same water temperature and photoperiod conditions as the adults in the facility. We made five replicates for each condition where approx. 30 larvae per tank were maintained throughout the treatment. We kept pairs of rearing tanks, i.e. one from each condition, into a white plastic box illuminated by LED strips (4000 K, White, Eglo).

From the pool of larvae that survived during the treatment, we randomly selected a total of 60 larvae from the enriched condition and 60 larvae from the barren condition. Behavioural observations were performed at three developmental stages, i.e. 7, 14, and 21dpf (*n* = 20 per subgroup). Before each observation test, individuals were randomly selected from one of the five replicates of each condition. Each larva was tested only once and subsequently transferred to standard post-test housing tanks for breeding purposes. All the living larvae that were not tested for this study were transferred to an isolated tank for breeding purposes.

### Novel object test

The experiment was conducted in a dark room to reduce external visual interference. The temperature and light conditions were equal to the ones provided during the treatment. The experimental apparatus was a 3D-printed rectangular tank (7 × 4 × 5 cm; Fig, 1c) made of white PLA material and filled with 90mL of Fish Water 1x. The stimulus, a 3D-printed black cone (0.7 cm base diameter, 1 cm height; Fig. 1d) was placed on a white pedestal (1.3 cm height) and positioned in one half of the apparatus. A previous study showed that this stimulus elicited in the subjects a neophobic response (Bruzzone et al. 2020). A Canon LEGRIA HFR38 video camera was positioned 90 cm above the apparatus to record the behavioural test. The experimental apparatus was placed into a white plastic box illuminated by LED strips (4000 K, White, Eglo). The position of the experimental apparatus into the white box, the height and zoom of camera were maintained fixed to acquire equal-quality video recording used for the following manual video analysis. However, to reduce the effect of spatial bias on behavioural response, we randomly alternate the position of the stimulus. The recording started from introducing the larva into the tank and continued for 10 min.

From the total sample of larvae survived, we randomly selected subjects to perform the behavioural assays at prescheduled age. For each test, a single larva at the specific developmental stage was gently transferred from its rearing tank to the experimental apparatus using a Pasteur pipette. Each larva was introduced into the centre of the empty half of the tank and was allowed to freely explore the apparatus and the novel stimulus inside. After the completion of each test, the water in the apparatus was replaced to ensure consistent test conditions between subjects.

### Video analysis

**Fig. 2:**
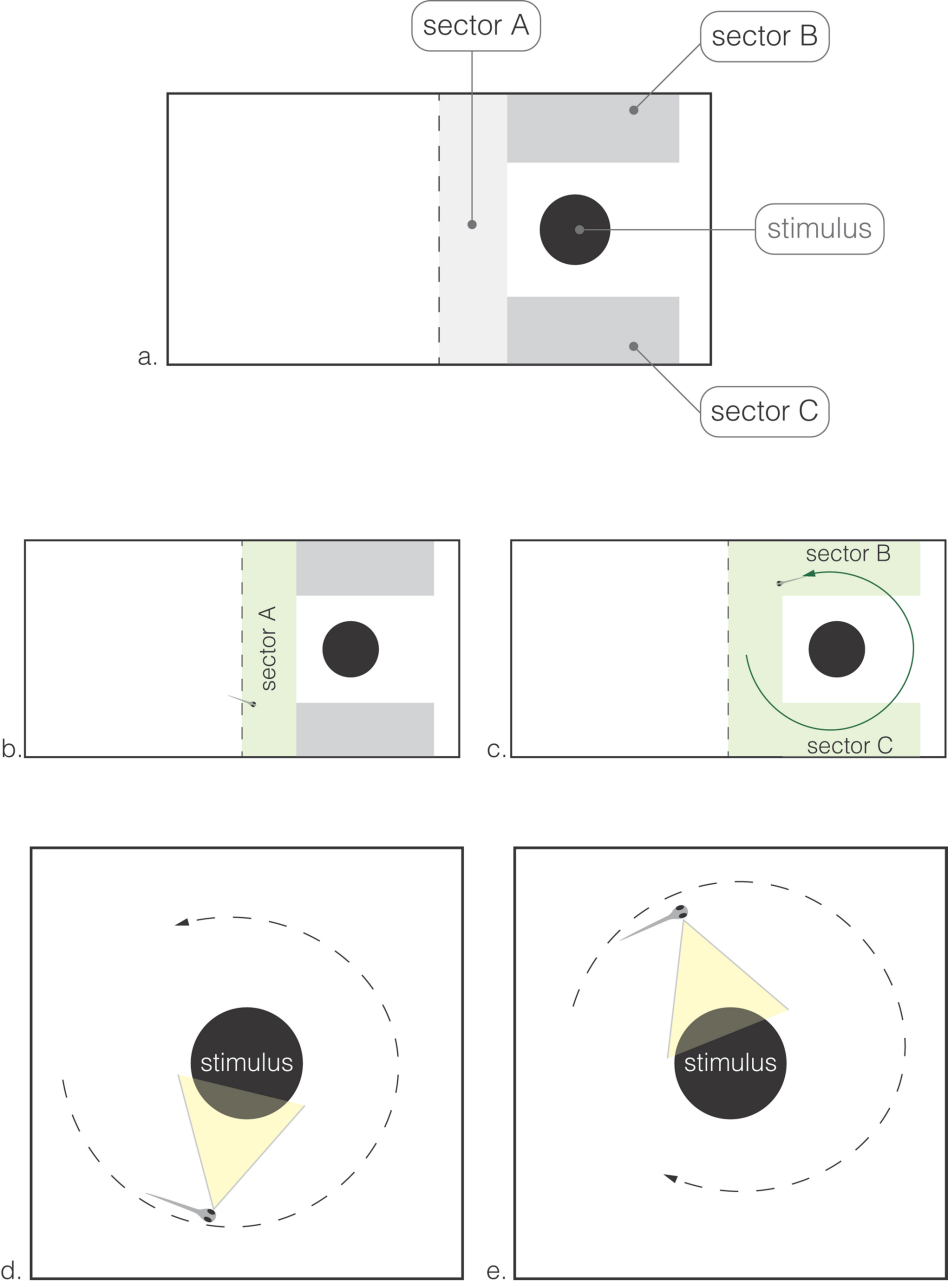
Schematic representation of the experimental apparatus and lateralized inspection behaviours. **a** Top view of the test tank showing the virtual subdivision of the half-sector containing the stimulus into three areas: Sector A (entry area), Sector B (area for right-eye observation), and Sector C (area for left-eye observation). **b** Larva entering Sector A, showing the “approach” behaviour. **c** Larva performing a completed rotation (i.e., inspection), in this example with the left eye. **d** Larva performing a counterclockwise inspection, observing the stimulus with its left eye. **e** Larva performing a clockwise inspection, observing the stimulus with its right eye

To assess the neophobic responses shown by larvae towards the novel stimulus, the half-sector containing the stimulus was virtually subdivided into three sectors (see Fig. 2a). Sector A represented the initial entry area, while sectors B and C corresponded to the areas that the subject could explore to further investigate the stimulus by using the right or left eye, respectively. The manual scoring was performed by an experimenter blind to the aim of the study.

The observer analysed the video recordings using purpose-designed software that allowed a transparent grid to be superimposed onto the video. This grid contained the three predefined sectors (A, B, and C), ensuring that the same spatial reference was consistently applied across all videos.

*Behavioural response*. We manually scored the video recordings to evaluate and quantify the behaviour of each subject. When subjects enter zone A, we classified the response as approach. We scored the total number of approaches and measured the time that subject needed to make their first approach.

*Lateralized response*. When subjects enter the zone A and performed a complete rotation around the stimulus starting from the zone B or C and ending at the opposite zone, we classified it as lateralized inspection. This response could be either clockwise (enter the sector B, use of the right eye to look at the stimulus and complete the rotation entering the sector C; Fig. 2c) or counterclockwise (enter the sector C, use of the left eye to look at the stimulus and complete the rotation entering the sector B; Fig. 2b). The lateralized responses resembled how individuals acquired information and differently split the processing between the two hemispheres (Miletto Petrazzini et al. 2020). From this series of lateralized inspection made by each subject, we calculated a lateralization index (*L*_*R*_) according to the following formula:

*index* = (Clockwise turns - Counterclockwise turns)/(Clockwise turns + Counterclockwise turns).

The relative lateralization index reflected the directionality of hemispheric processing, whereas the absolute value considered the degree of hemispheric processing regardless of directionality.

### Statistical analysis

Statistical analyses were conducted using RStudio, version 4.2.1 (2022-06-23 ucrt) (R Foundation for Statistical Computing, Vienna, Austria, https://www.r-project.org). The significance threshold was set at *P* < 0.05.

Behavioural (i.e., number of approached, time to perform the first approach) and lateralized responses (number of completed lateralized approaches, relative and absolute lateralization index) were analysed with a similar approach. The effect of treatment on the behavioural/lateralized variable response and its potential differed effect among ages were analysed via linear effects models (“lme4” Rpackage; Bates et al. 2015) fitted with treatment condition (enrichment vs. barren) and age (three levels factors: 7-, 14-, 21-dpf) as fixed effect. The significance of model’s parameters was assessed via Type 3 tests performed with ‘Anova’ function from the ‘car’ Rpackage. The number of completed lateralized rotations (i.e., inspections) was square-root transformed before being analysed to increase model fitting. All other considered response variables showed a normal distribution and the statics inferences followed nothe model analysis. Both models used for analysing the lateralization indices were additionally fitted with the square-root transformed number of inspections to increase model fitting. Given the implication of a triple interaction involving a covariate (age × treatment × number of inspections), we performed model selection from the full model via the “stepAIC” function from the “MASS” R packaged. The model selected by AIC was used for assessing differences in the response variables among groups. For the relative lateralization index, the selected model was the full model without the triple interaction treatment × age × number of completed rotation. For the absolute lateralization index, the selected model was fitted with treatment as fixed factor and the number of completed rotation as covariate. No interaction term was included in this model. Subjects that did not display a completed lateralized responses (total number: 12) were excluded from the model. We also tested whether the distribution of the relative lateralization index for each group was normally distributed using One-sample Kolmogorov-Smirnov test via the ‘ks.test’ function (argument ‘pnorm’).

To further explore the role of treatment in shaping the behaviour – lateralization covariance, we performed correlation analysis between the two behavioural traits (number of approaches and time to make the first approach) and the two lateralized indices (relative and absolute). Due to the large number of correlation analyses performed (age × treatment × 4 traits covariation), we performed Bonferroni multiple correction on p-values. To further explore the role of treatment in shaping this relationship, we conducted an additional analysis described in the Behaviour – Lateralization Covariance section.

## Results

### Neophobic response towards the novel stimulus

**Fig. 3:**
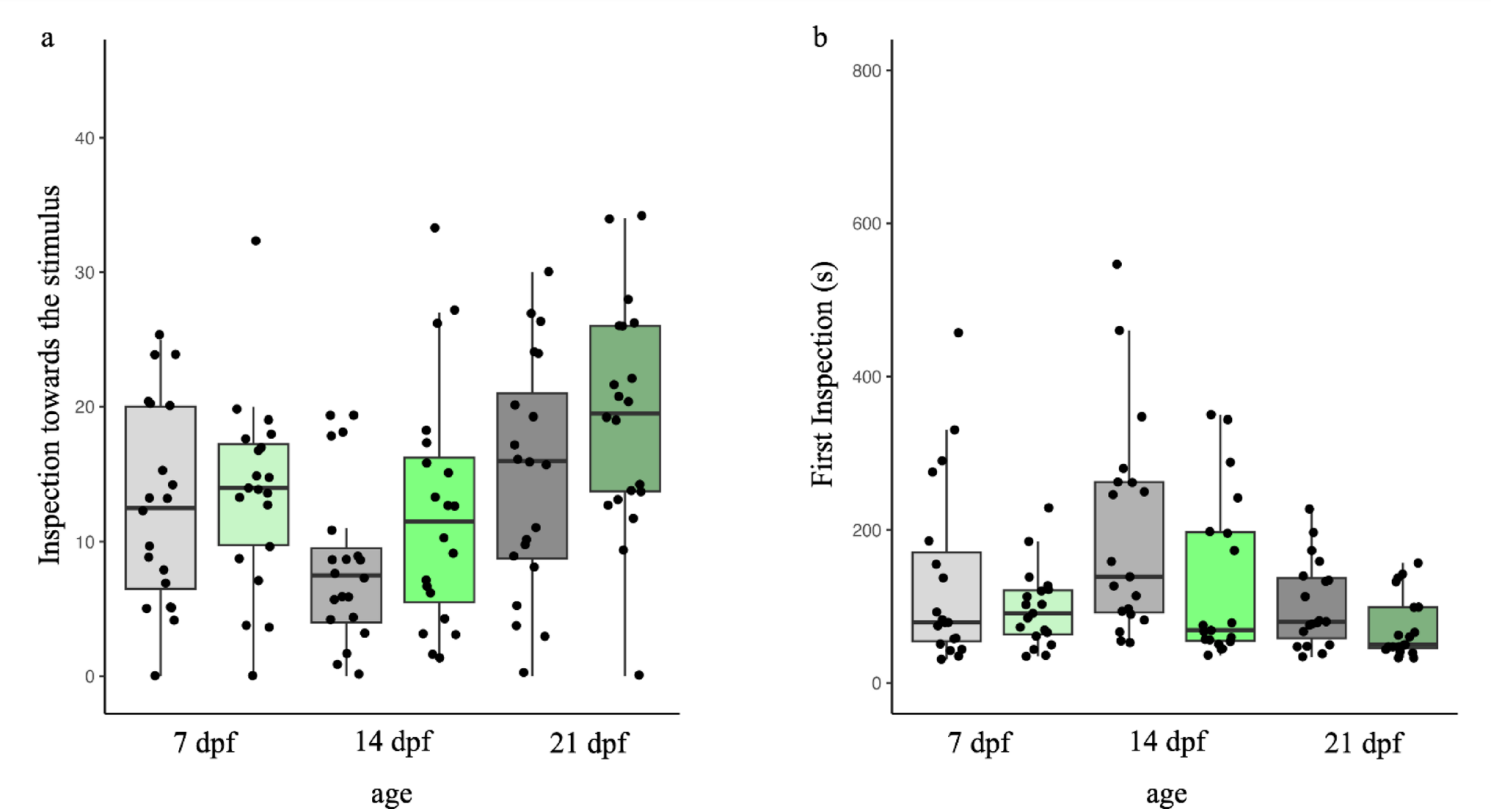
**a** Number of inspections towards the novel stimulus and **b** latency to the first approach performed by the 7-, 14-, and 21-dpf larvae divided per treatment condition, i.e. grey colours: larvae exposed to the barren environment; green colours: larvae exposed to the enriched environment

Considering the overall number of approaches toward the stimulus, the analysis revealed a significant effect of treatment (χ^2^_1_ = 4.729, *P* = 0.032, η^2^_p_ = 0.04), indicating that larvae exposed to the enriched environment performed more approaches (15.03 ± 8.58) compared to larvae from the barren condition (11.93 ± 7.80) (Fig. 3a). The analysis also revealed a significant difference in the number of approaches among ages (χ^2^_1_ = 7.528, *P* < 0.001, η^2^_p_ = 0.12). Post hoc tests revealed that 21dpf larvae performed more approaches than 14dpf (*P* < 0.001) and 7dpf (*P* = 0.072) larvae, but no differences emerged between 7dpf and 14dpf larvae (*P* = 0.231). No interaction effect was significant (age × treatment: χ^2^_1_ = 0.569, *P* = 0.568, η^2^_p_ > 0.01).

Larvae from the enriched condition made on average a faster first approach towards the stimulus (100.32 ± 75.01s) compared to larvae from the barren condition (144.68 ± 116.39s) (χ^2^_1_ = 6.233, *P* = 0.014, η^2^_p_ = 0.05; Fig. 3b). A significant effect of age was also found (χ^2^_1_ = 5.359, *P* = 0.006, η^2^_p_ = 0.09). Post hoc tests revealed that 21dpf larvae were faster in inspecting the stimulus compared to 14dpf larvae (*P* = 0.004) but not compared to 7dpf larvae (*P* = 0.129), and there were no differences between 7dpf and 14dpf larvae (*P* = 0.400). No interaction effect emerged as significant (age × treatment: χ^2^_1_ = 0.650, *P* = 0.523, η^2^_p_ = 0.01).

### Lateralized inspection towards the novel stimulus

Considering the number of lateralized inspections, the analysis revealed no significant effect of treatment (χ^2^_1_ = 2.500, *P* = 0.117, η^2^_p_ = 0.02), no significant differences among ages (χ^2^_1_ = 2.667, *P* = 0.074, η^2^_p_ = 0.04), and no interaction (χ^2^_1_ = 0.557, *P* = 0.574, η^2^_p_ > 0.01), suggesting that larvae of different ages and from different treatment conditions showed similar lateralized responses towards the stimulus (Fig. S1).

**Fig. 4:**
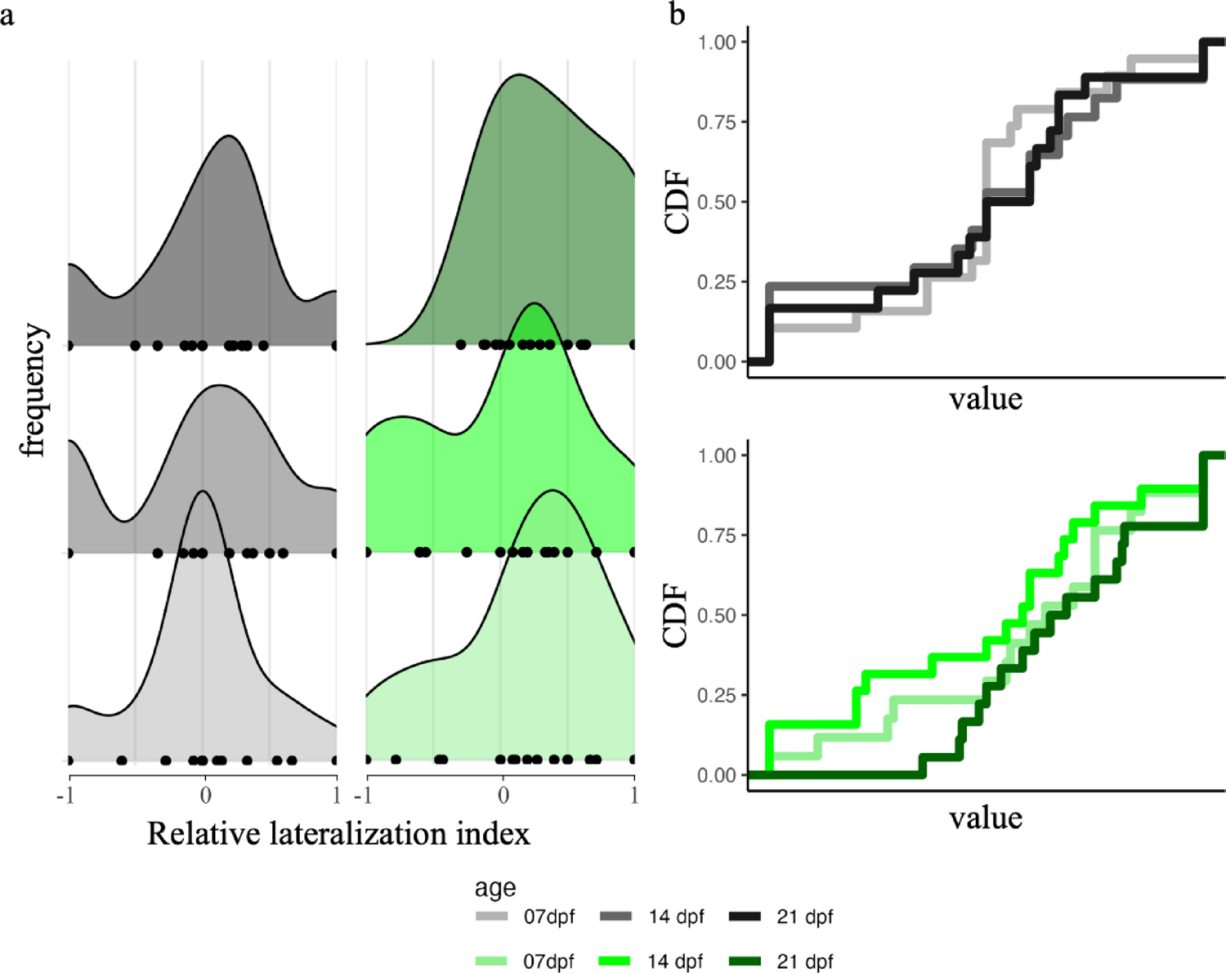
**a** Frequency distribution of the relative lateralization index divided per ages (7-, 14-, and 21-dpf) and, i.e. grey colours: larvae exposed to the barren environment; green colours: larvae exposed to the enriched environment. The black point in panel represented the score per individuals. Cumulative distribution function of larvae exposed to the **b** barren condition or **c** environmental enrichment divided per ages

The frequency distribution of the relative lateralized index, divided by ages and treatment, is represented in the Fig. 4a. From these distributions, we observed that all groups, except for the 21dpf larvae exposed to environmental enrichment, did not show a lateralized bias at the group level (all One-sample Kolmogorov-Smirnov test Ps > 0.05). This pattern likely reflects the individual variation of inspecting the novel object using the right-eye (right-tail of distribution; values close to 1) or left-eye (left-tail of distribution; value close to −1). In contrast, 21dpf larvae exposed to environmental enrichment showed a preferred right-eye use (right-tail of the distribution, i.e. values close to 1; One-sample Kolmogorov-Smirnov test: D = 0.395, *P* = 0.007). However, when all data were grouped, no statistically significant bias for right or left inspections was found at the population level (one sample t-test: *t*_107_ = 1.568, *P* = 0.120). From the cumulative distribution plot divided by treatment (Fig. 4b), barren larvae of the three ages showed a crossing point at 0.5 on the y-axis, indicating similar medians. Conversely, larvae from the enriched condition showed different overlap of their cumulative distribution (Fig. 4c): 14dpf larvae showed higher variance compared to other ages, i.e. the black line is above the other lines on the left of crossing point and below on the right, whereas 21dpf larvae showed lower variance of their lateralized response. The analysis revealed a marginally no-significant effect of treatment on relative lateralized index (χ^2^_1_ = 3.791, *P* = 0.054, η^2^_p_ = 0.04), however, a significant interaction age × completed rotation effect emerged (χ^2^_1_ = 3.791, *P* = 0.054, η^2^_p_ = 0.04) suggesting that the relationship between the lateralized inspection and the strength of novel stimulus’ exploration, i.e. the number of completed rotation, differed among ages. Pos hoc tests on the trend revealed that 21dpf larvae a deeper negatively trend of this relationship compared to 14dpf (*P* = 0.045) but not compared to 7dpf larvae (*P* = 0.915), and there were no differences between 7dpf and 14dpf larvae (*P* = 0.245). No main effects or interactions emerged as significant (Ps > 0.095).

**Fig. 5:**
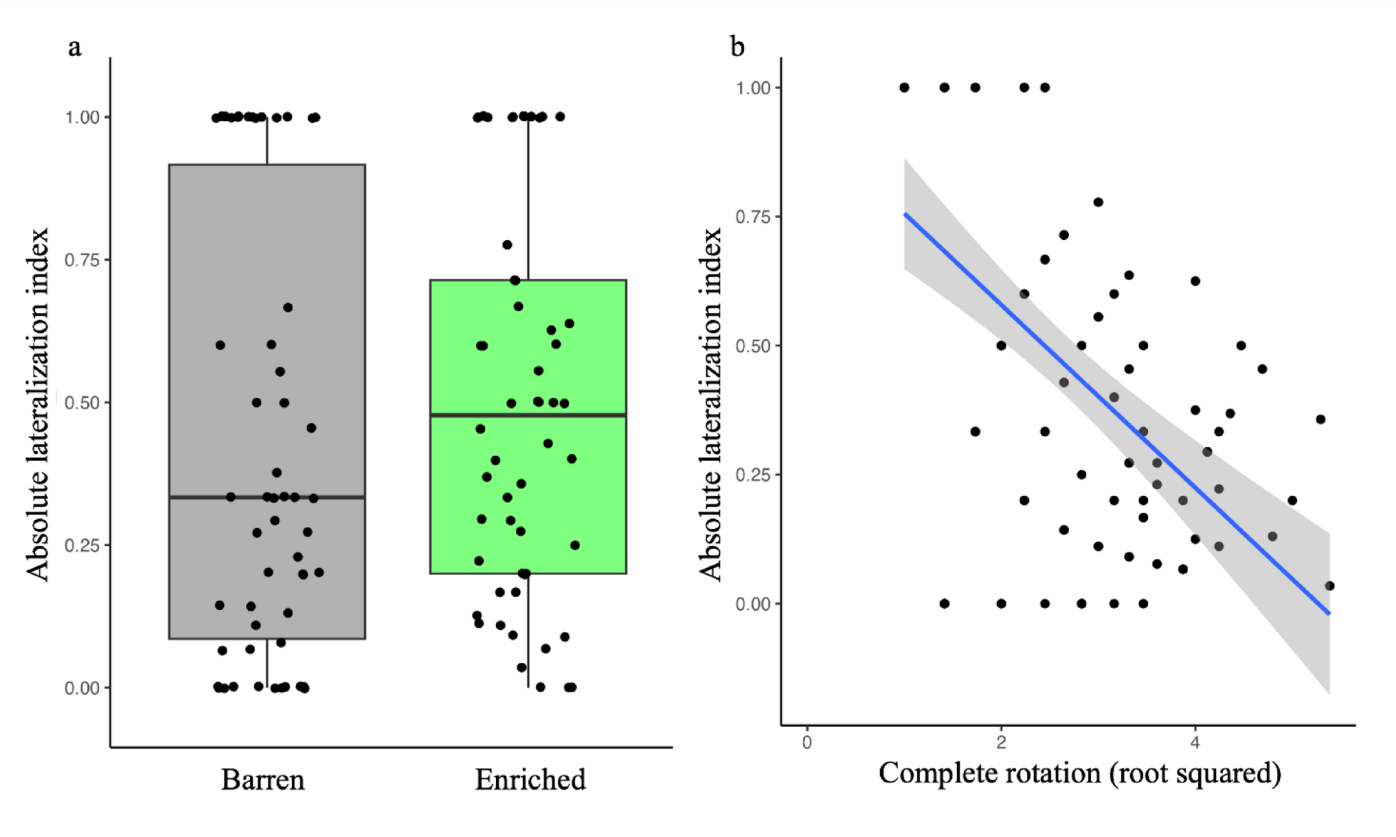
The graphs represented the revealed significant main effects of condition **a** and number of **b** completed rotation on the absolute lateralization index. In the graph **a**, the two colours represented the environmental conditions, i.e. grey colours: larvae exposed to the barren environment; green colours: larvae exposed to the enriched environment

Considering the absolute lateralization index, the analysis revealed a significant effect of treatment (χ^2^_1_ = 6.435, *P* = 0.013, η^2^_p_ = 0.06; Fig. 5a), indicating that larvae exposed to an enriched environment showed a stronger degree of lateralization (0.49 ± 0.34) compared to larvae exposed in a barren environment (0.42 ± 0.38). The degree of lateralization was significantly negatively predicted by the number of lateralized inspections (χ^2^_1_ = 50.145, *P* < 0.001, η^2^_p_ = 0.35; Fig. 5b), suggesting that more active larvae in exploring the stimulus showed a weaker degree of lateralization. The selected model did not include age as fixed factor, suggesting that treatment, rather than the developmental stages of larvae, significantly shaped the lateralized response.

### Behaviour – Lateralization covariance analysis

**Fig. 6:**
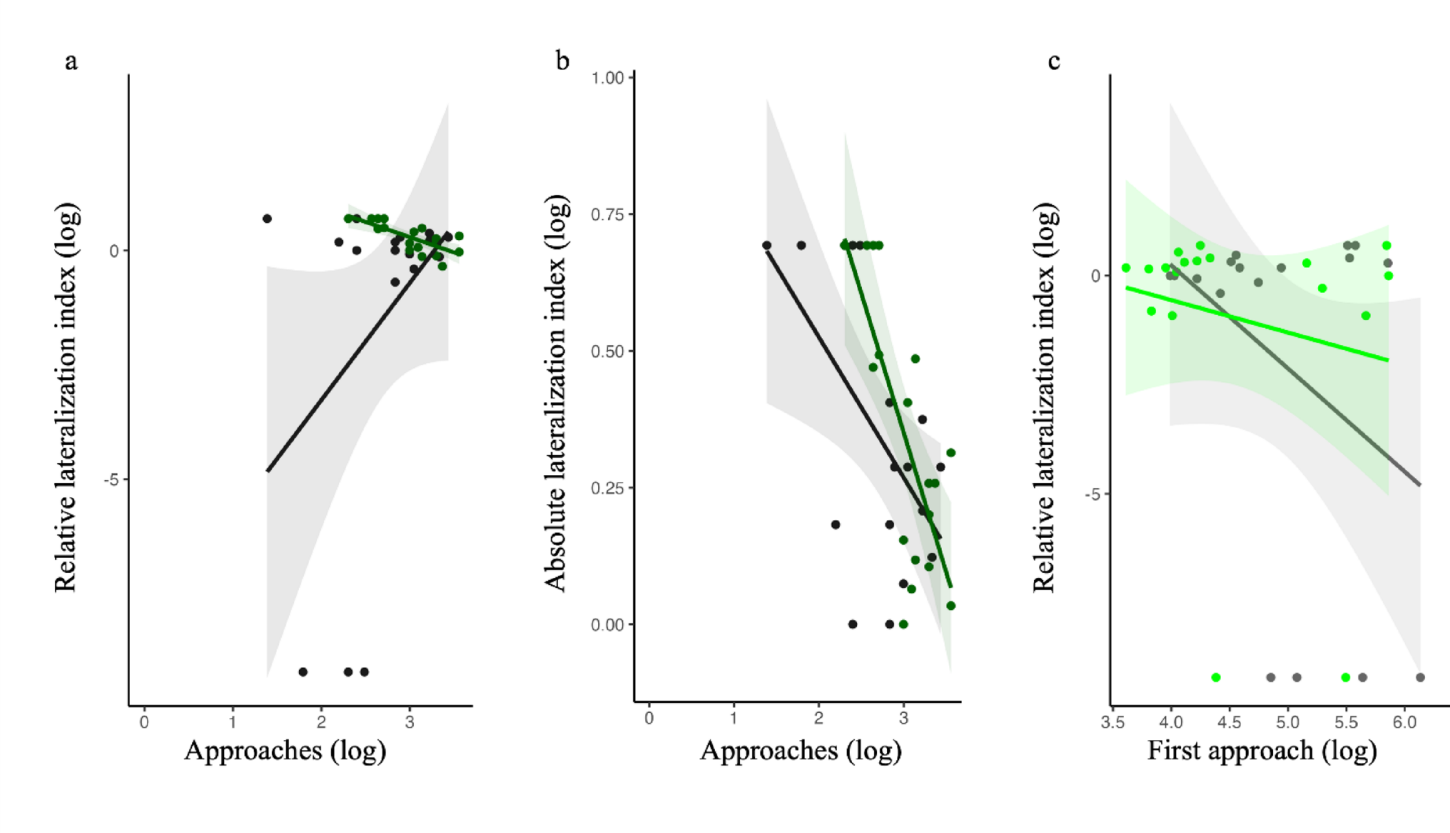
Scatterplots representing the covariation between the behavioural trait (graph **a** and **b**: approaches, graph **c**: latency to the first approach) and the two lateralization traits (graph **a** and **c**: relative lateralization index; graph **b**: absolute lateralization index) in the barren (grey colour) and enriched (green colour) larvae

The covariance analysis suggested that treatment significantly alter the correlation between behavioural and lateralized responses in the 21dpf larvae. In the enriched group, the number of inspections negatively predicted both the direction of lateralization (Pearson *r* =−0.732, *t*_*16*_ = 4.304, *P* = 0.013; Fig. 6a) and the degree of lateralization (Pearson *r* =−0.741, *t*_*16*_ = 4.419, *P* = 0.010; Fig. 6b). In contrast, larvae from the barren condition did not show these significant covariations (number of inspections and relative lateralized index: Pearson *r* = 0.398, *t*_*16*_ = 0.102, *P* > 0.999; Fig. 6a; number of inspections and absolute lateralized index: Pearson *r* = −0.575, *t*_*16*_ = 2.814, *P* = 0.300; Fig. 6b). Additionally, in 14dpf larvae from the barren condition, the latency to the first approach correlated with the degree of lateralization (Pearson *r* = 0.716, *t*_*16*_ = 3.972, *P* = 0.029; Fig. 6c), indicating that individuals more willing to explore the stimulus showed a weaker absolute lateralization index. This covariation was absent in the larvae of the same age but raised in the enriched environment (Pearson *r* = 0.163, *t*_*16*_ = 0.662, *P* > 0.999; Fig. 6c). No other correlations were significant (Table S1).

## Discussion

Results of this study indicate that the environmental conditions experienced by individuals during early life stages shape phenotypic variation of neophobic responses in zebrafish larvae. Despite age-related differences were less pronounced in the behavioural and lateralization measures that we considered for studying the neophobic response, the influence of early exposure to enrichment greatly affected older larvae. Together, these findings support our initial hypothesis that environment conditions influence neophobia, the degree of lateralization, and the covariance of behavioural traits, which can potentially be detrimental for fitness.

Previous studies have primarily investigated neophobia by comparing individuals at broadly distinct life stages (e.g., juveniles versus adults), potentially overlooking more subtle developmental transitions. In species characterized by rapid ontogeny, such as zebrafish, it is plausible that individual differences in neophobic behaviour may emerge during early larval stages, when environmental experience exerts a stronger influence on behavioural and cognitive development (Crane and Ferrari 2017). Our present findings support this hypothesis and extend previous literature by demonstrating that environmental experiences influence several important aspects of zebrafish (Lee et al. 2019; Gatto et al. 2024), and potentially, the environment might be a key driver in shaping neophobic phenotype during the early stage of life. As shown in previous studies, environmental enrichment during zebrafish development enhances their survivorship, cognitive and emotional flexibility, promoting exploratory behaviours and reducing anxiety-like responses (von Krogh et al. 2010; Gatto et al. 2022). Our results supported that 21dpf larvae raised in enriched environments not only approached novel stimuli more frequently, but also initiated their first approach more rapidly than age-matched controls from barren conditions.

As outlined above, age-related differences in neophobic behaviour were observed in the present study. However, contrary to previous findings, we did not detect a significant interaction between treatment condition and developmental stage. This result suggests that the influence of environmental enrichment on neophobic responses may not be uniform across development, but rather may vary depending on the specific stage of larval maturation. In other words, while treatment conditions exert a modulatory effect on neophobic behaviour, the magnitude and direction of this effect appear to be developmentally dependent. One possible explanation for this discrepancy with the existing literature lies in the methodological differences between studies. The present work employed a distinct set of behavioural measures, specifically designed to provide a more detailed characterization of neophobic responses, which intentionally diverge from the more conventional measures previously adopted in similar research on zebrafish larvae. However, despite the absence of a statistically significant interaction between age and treatment condition, the directionality of the observed effects remains consistent with that reported in earlier studies, thereby supporting the broader conclusion that both age and treatment contribute meaningfully to behavioural development in zebrafish. Despite the ongoing evidence supporting the application of rearing protocol based on environmental enrichment (Volgin et al. 2018; Gallas-Lopes et al. 2023), its influence in several aspect of zebrafish life remains largely unexplored and highlights the need for further empirical investigation.

When examining the results related to lateralization, two main observations emerge. First, in terms of directionality, the relative lateralization index did not reveal any significant preference for the use of the right or left eye, and this pattern generally remained stable across developmental stages. However, the absolute lateralization index revealed that larvae from the enriched condition exhibited a stronger degree of lateralization when exploring the novel stimulus compared to those from the barren condition. Overall, we observed notable individual differences in both direction and degree of lateralization, which appeared to diminish with increasing age. Especially, early-life stage larvae (7 and 14dpf) showed larger dispersion of visual lateralized inspection towards the novel stimulus compared to the older stages, which showed a right-eye use preference for inspecting the novel stimulus. This finding suggests that exposure to an enriched environment may reduce inter-individual variability in lateralization, a phenomenon observed in amphibian and other vertebrates (Bisazza and Lucon-Xiccato 2025). However, our study did not evaluate the persistence of individual differences in lateralization across life-stages, which required further attention for its adaptive relevance for the maintenance of a genotype (Frasnelli and Vallortigara 2018).

The most robust and meaningful effects were observed in relation to the degree of lateralization, which was significantly enhanced by environmental enrichment. This result may be attributable to the increased sensory demands associated with the enriched condition, which require individuals to process a plethora of information through improved cognitive capacity, thereby increasing their chance of survival (Vallortigara and Rogers 2005). Similar findings have been reported in the literature, where early-life experiences of environmental complexity have been shown to modulate the expression of lateralization. For instance, Brown and colleagues (2007) showed that fish from high-predation areas were more lateralized than those from low-predation areas while Bibost and colleagues (2013) investigated the influence of environmental complexity on the development of brain lateralization and found that rearing conditions influenced the development of laterality in each sex differently. These findings support the hypothesis that the complexity of information derived from the environment during early development plays a crucial role in shaping the degree of functional asymmetry in the brain.

While we can assume that the environment influences lateralization consistently across developmental stages, we secondarily found that not only was the average degree of lateralized inspections affected by early experience to enrichment, but so were individual differences, particularly the correlation between lateralized response and neophobic response. Individual differences in lateralization can be expressed as a result of phenotypic plasticity in an adaptive way, since environmental factors can shape both the direction and strength of lateralized behaviour (Bisazza and Lucon-Xiccato 2025). To note, we observed that individual differences in neophobic response predict visual lateralized inspection particularly in 21dpf larvae, whereas it is not observed in the other two age groups. It is possible that this finding is, at least in part, attributable to the longer duration of exposure to enrichment stimuli in these individuals. Therefore, we cannot rule out the possibility that developmental stage and exposure duration interacted to produce this outcome. Nonetheless, previous work by Gatto et al. (2022) demonstrated that even brief exposure to an enriched environment was sufficient to elicit a behavioural response, suggesting that the impact of enrichment may emerge rapidly during early development. Once again, it cannot be excluded that methodological differences between the study by Gatto et al. (2022) and the present work may highlight distinct aspects of the phenomenon; however, these differences do not compromise the overall coherence of the broader pattern observed across studies. The present study was limited to the first 21dpf, which correspond to the last week of larval phase before juveniles in zebrafish development (Schilling 2002; Westerfield 2007). While this may be considered a limitation, as later developmental stages (juvenile and adult) were not included, it should be noted that the rapid developmental trajectory of this species makes the first 21 days of life largely predictive of subsequent phenotypic outcomes (Fero et al. 2011). Therefore, although caution is warranted when extrapolating beyond the larval phase, the findings presented here provide meaningful insights into early developmental processes.

The interplay between habitat type and the covariance of personality and cognition has become increasingly recognized as a crucial aspect of behavioural ecology. Variability in habitat complexity and ecological pressure can influence not only individual behavioural tendencies but also how these traits co-vary with cognitive performance. For instance, De Meester and colleagues (2022) tested the effect of habitat complexity on multiple aspects of personality and cognition in the Aegean wall lizard (*Podarcis erhardii*) and found that lizards from more complex habitats performed better on a spatial learning task even tough individuals from open (simple) environments exhibit a stronger association between boldness and cognitive performance. In zebrafish, early-life exposure to environmental complexity, including structural enrichment or predator cues, has been shown to influence both lateralization and learning performance, thereby affecting how personality traits such as boldness or neophobia covary with cognitive traits (DePasquale et al. 2016; Lucon-Xiccato et al. 2017). For instance, Lucon-Xiccato et al. (2017) demonstrated that prenatal exposure to predator stimuli enhances predator recognition learning through increased hemispheric specialization, suggesting that habitat-mediated stressors can alter brain asymmetries and their behavioural correlates. These findings highlight the plastic and context-dependent nature of the personality-cognition relationship, indicating that environmental inputs during critical developmental windows can reshape individual cognitive styles and their alignment with personality traits. In zebrafish, which exhibit high developmental plasticity, such covariance may be especially sensitive to early environmental conditions. From a broader perspective, these findings contribute to the understanding of how early environmental experiences shape behavioural phenotypes in a teleost displaying an extreme altriciality-like developmental mode. Neophobia and lateralization are traits that can influence survival, dispersal, and resource exploitation in natural populations. The observed link between enrichment, neophobia, and lateralization provides insights into how environmental complexity fosters behavioural and cognitive diversity. Our findings supported the importance of considering ontogenetic experience on fish behaviour in both basic research and applied contexts, such as improving animal welfare, conservation breeding, and reintroduction programs (Stevens et al. 2021; Arechavala-Lopez et al. 2022).

## Supplementary Information

Below is the link to the electronic supplementary material.

Supplementary Material 1

## Data Availability

Data supporting the conclusions of this article are provided by the corresponding author upon request.
