# Supplementary material for: Environmental enrichment influences the relationship between lateralization and neophobia in zebrafish larvae
Source: Anim Cogn. 2025 Dec 24;29(1):3. doi: 10.1007/s10071-025-02025-1 (PMC12738612; doi:10.1007/s10071-025-02025-1)
Supplement: Supplementary file 1 — Supplementary Material 1 [file 10071_2025_2025_MOESM1_ESM.docx]

**Environmental enrichment influences the relationship between lateralization and neophobia in zebrafish larvae**

Gabriela Gjinaj^1*^, Elia Gatto^2,3*^ and Marco Dadda^1^

^1^ Department of General Psychology, University of Padova, Via Venezia 8, 35131 Padova (Italy)

^2^ Department of Chemical Pharmaceutical and Agricultural Sciences, University of Ferrara, Via Luigi Borsari 46, 44121 Ferrara (Italy)

^3^ Department of Life Sciences and Biotechnology, University of Ferrara, Via Luigi Borsari 46, 44121 Ferrara (Italy)

*Corresponding authors: Gabriela Gjinaj, [gabriela.gjinaj@unipd.it](mailto:gabriela.gjinaj@unipd.it); Elia Gatto, [elia.gatto@unife.it](mailto:elia.gatto@unife.it)

**Figures S1**

**Tables S1**

**Figure S1.**  Number of completed lateralized rotation performed by the 7-, 14-, and 21-dpf larvae divided per treatment condition, i.e. grey colours: larvae exposed to the barren environment; green colours: larvae exposed to the enriched environment.


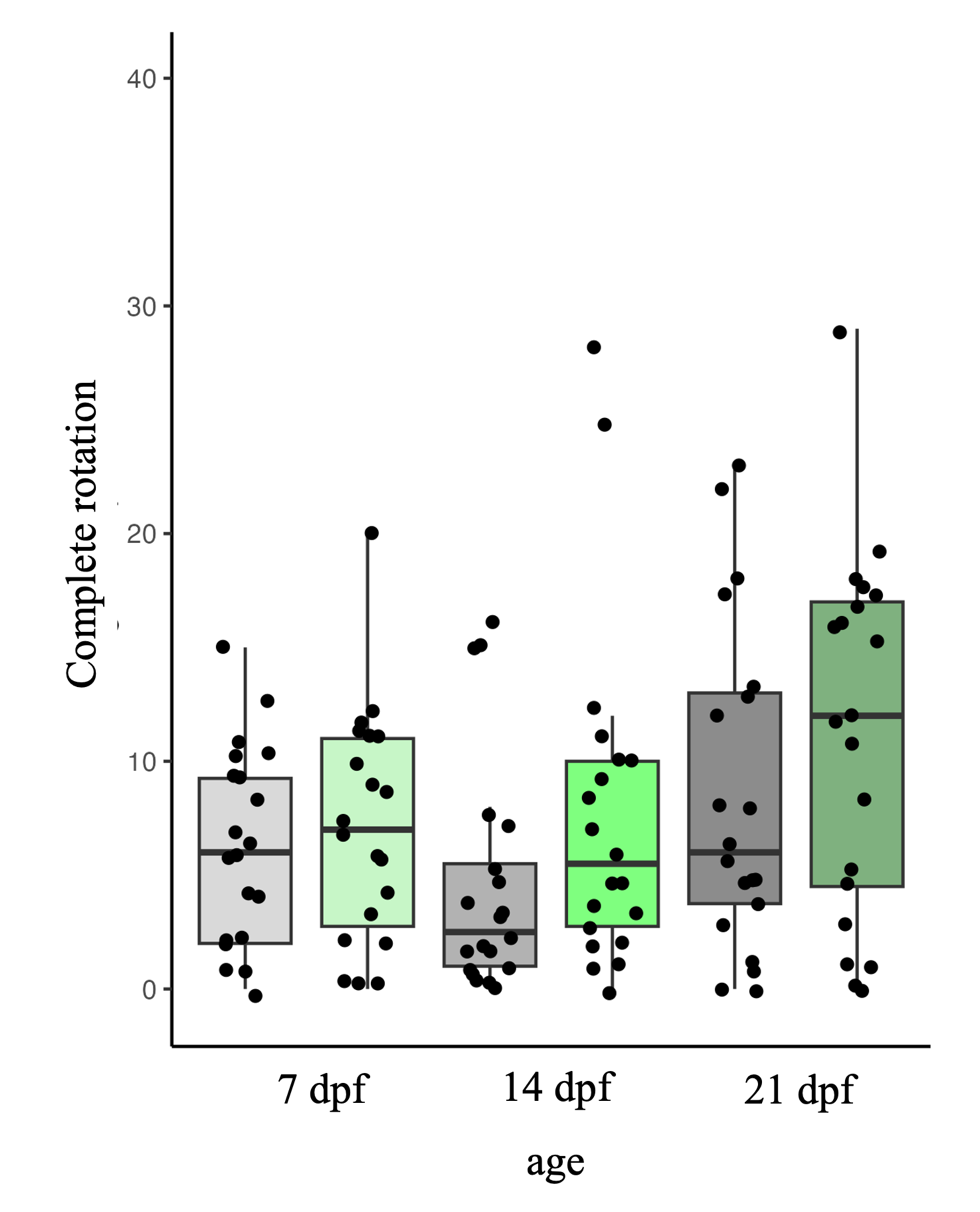


**Table S1**

Pearson Product-Moment Correlations and Confidence Intervals of the behavioural and lateralization trait correlations. P values were corrected for multiple comparison (Bonferroni) to reduce type I error. Bold values indicate significant pairwise correlations between traits.

| condition | ages | Behavioural trait | Lateralization trait | Pearson-Moment Correlation | P value corrected for multiple comparison |
| --- | --- | --- | --- | --- | --- |
| Barren | 7 dpf | Inspection | Relative Lateralization Index | r = 0.14  *t*_17_ = 0.563 P = 0.581 | N.S. |
| Barren | 14 dpf | Inspection | Relative Lateralization Index | r = 0.46  *t*_15_ = 1.998 P = 0.064 | N.S. |
| Barren | 21 dpf | Inspection | Relative Lateralization Index | r = 0.40  *t*_16_ = 1.737 P = 0.102 | P = 0.300 |
| Enriched | 7 dpf | Inspection | Relative Lateralization Index | r = 0.18  *t*_15_ = 0.183 P = 0.857 | N.S. |
| Enriched | 14 dpf | Inspection | Relative Lateralization Index | r = 0.32  *t*_17_ = 1.391 P = 0.182 | P = 0.623 |
| Enriched | 21 dpf | Inspection | Relative Lateralization Index | r = -0.73  *t*_16_ = 4.304 P < 0.001 | **P = 0.010** |
| Barren | 7 dpf | First approach | Relative Lateralization Index | r = -0.11  *t*_17_ = 0.474 P = 0.641 | N.S. |
| Barren | 14 dpf | First approach | Relative Lateralization Index | r = 0.38  *t*_15_ = 1.572 P = 0.137 | **P = 0.029** |
| Barren | 21 dpf | First approach | Relative Lateralization Index | r = 0.40  *t*_16_ = 1.765 P = 0.097 | N.S. |
| Enriched | 7 dpf | First approach | Relative Lateralization Index | r = -0.02  *t*_15_ = 0.078 P = 0.939 | N.S. |
| Enriched | 14 dpf | First approach | Relative Lateralization Index | r = -0.19  *t*_16_ = 0.765 P = 0.455 | N.S. |
| Enriched | 21 dpf | First approach | Relative Lateralization Index | r = 0.32  *t*_16_ = 1.367 P = 0.191 | N.S. |
| Barren | 7 dpf | Inspection | Absolute Lateralization Index | r = -0.04  *t*_17_ = 0.183 P = 0.857 | N.S. |
| Barren | 14 dpf | Inspection | Absolute Lateralization Index | r = -0.39  *t*_15_ = 1.659 P = 0.118 | N.S. |
| Barren | 21 dpf | Inspection | Absolute Lateralization Index | r = -0.57  *t*_16_ = 2.814 P = 0.012 | N.S. |
| Enriched | 7 dpf | Inspection | Absolute Lateralization Index | r = -0.50  *t*_15_ = 2.218 P = 0.042 | N.S. |
| Enriched | 14 dpf | Inspection | Absolute Lateralization Index | r = -0.51  *t*_17_ = 2.439 P = 0.026 | N.S. |
| Enriched | 21 dpf | Inspection | Absolute Lateralization Index | r = -0.74  *t*_16_ = 4.419 P < 0.001 | **P = 0.013** |
| Barren | 7 dpf | First approach | Absolute Lateralization Index | r = -0.07  *t*_17_ = 0.272 P = 0.789 | N.S. |
| Barren | 14 dpf | First approach | Absolute Lateralization Index | r = 0.16  *t*_16_ = 0.662 P = 0.517 | N.S. |
| Barren | 21 dpf | First approach | Absolute Lateralization Index | r = 0.18  *t*_16_ = 0.732 P = 0.475 | N.S. |
| Enriched | 7 dpf | First approach | Absolute Lateralization Index | r = 0.05  *t*_15_ = 0.186 P = 0.855 | N.S. |
| Enriched | 14 dpf | First approach | Absolute Lateralization Index | r = 0.16  *t*_16_ = 0.662 P = 0.517 | N.S. |
| Enriched | 21 dpf | First approach | Absolute Lateralization Index | r = 0.30  *t*_16_ = 1.249 P = 0.230 | N.S. |
